# Effectiveness of an evidence-based chiropractic continuing education workshop on participant knowledge of evidence-based health care

**DOI:** 10.1186/1746-1340-14-18

**Published:** 2006-08-24

**Authors:** Ronald J Feise, Jaroslaw P Grod, Anne Taylor-Vaisey

**Affiliations:** 1 Institute of Evidence-Based Chiropractic, 6252 Rookery Road, Fort Collins, Colorado, USA; 2 Canadian Memorial Chiropractic College, 6100 Leslie Street, Toronto, Ontario, Canada

## Abstract

**Background:**

Chiropractors must continue to learn, develop themselves professionally throughout their careers, and become self-directed and lifelong learners. Using an evidence-based approach increases the probability of optimal patient outcomes. But most chiropractors lack knowledge and interest in evidence-based approaches. The purpose of this study was to develop and measure the effectiveness of evidence-based training for chiropractic practitioners in a continuing education setting.

**Methods:**

We developed and evaluated a continuing education workshop on evidence-based principles and methods for chiropractic practitioners. Forty-seven chiropractors participated in the training and testing. The course consisted of 12.5 hours of training in which practitioners learned to develop focused questions, search electronic data bases, critically review articles and apply information from the literature to specific clinical questions. Following the workshop, we assessed the program performance through the use of knowledge testing and anonymous presentation quality surveys.

**Results:**

Eighty-five percent of the participants completed all of the test, survey and data collection items. Pretest knowledge scores (15-item test) were low (47%). Post intervention scores (15-item test) improved with an effect size of 2.0. A 59-item knowledge posttest yielded very good results (mean score 88%). The quality of presentation was rated very good, and most participants (90%) would "definitely recommend" or "recommend" the workshop to a colleague.

**Conclusion:**

The results of the study suggest that the continuing education course was effective in enhancing knowledge in the evidence-based approach and that the presentation was well accepted.

## Background

Society assumes that all health care professionals will continue to learn and develop themselves professionally throughout their lifetimes [1]. Practitioners spend numerous hours each year in continuing education (CE) programs that ostensibly focus upon improving management performance and/or optimizing patient outcomes [2]. CE events are intended to bring health professionals up-to-date with rapidly expanding medical information. Moreover, CE educators have a duty to provide information that has an affirmative impact on the health of patients (e.g., safe and effective).

Using an evidence-based approach increases the probability of optimal patient outcomes by improving the health care decision process [3,4]. Chiropractic has, for the most part, relied on knowledge based on anecdote and tradition. Diagnostic and therapeutic intervention decisions are often made with little to no attention to evidence-based methods. Instruction in evidence-based methods by chiropractic college clinics is almost absent [5]. However, a recent survey of current curricula of chiropractic colleges found most colleges to be teaching evidence-based concepts [6].

Chiropractors are ambivalent about the potential value of the evidence-based approach in patient care. Some think the approach is more academic than practical, and most lack knowledge about evidence-based health care (EBHC) [7,8]. The increasing trend towards health care provider accountability demands that chiropractors have the essential competencies to perform effectively and efficiently in a highly competitive health care system [9]. Because of the exponential growth of information, practitioners must acquire new skills to translate mountains of data into improved patient results. Although a wide range of scientific studies has been critically appraised in review articles, practitioners still need a knowledge of EBHC principles before they can properly apply the information from these articles to patient management [10].

Reports about teaching the evidence-based approach in the medical profession are fairly common [11-20]. Taylor reviewed medical education studies and found evidence that teaching critical appraisal skills improves both knowledge and attitudes towards evidence-based decision making [21]. Reviews by Davis found interactive medical educational interventions to be generally effective in changing practitioner behavior and even in improving patient outcomes [2,22,23].

In contrast, published information on EBHC education in the chiropractic field is rare. Five uncontrolled studies reported improved critical appraisal skills and attitudes towards an evidence-based approach among chiropractic students [24-28]. However we found no published studies of an educational intervention focusing upon teaching EBHC methods to chiropractic practitioners. If chiropractors are to become effective evidence-based practitioners, they need focused educational programs targeting specific competencies. Acknowledging this need, we developed a workshop for practitioners. This workshop teaches each of the fundamental skills required for practicing evidence-based health care.

Our objective was to develop and measure the effectiveness of evidence-based training for chiropractic practitioners in a continuing education setting. Does an evidence-based workshop result in improved knowledge of evidence-based procedures? How do participants rate the quality of the presentation?

## Method

### Overview

This study was an uncontrolled before-after design. We developed an interactive evidence-based workshop for chiropractic practitioners in a continuing education setting. We then measured participants' knowledge of the evidence-based approach with a fifteen-item pretest-posttest and a 59-item post-intervention measure. We also collected data regarding participant demographics and their perception of the presentation.

### Setting and sample

Participants were chiropractic practitioners enrolled in the third year of a 300-hour 3-year Chiropractic Rehabilitation Sciences program through the Canadian Memorial Chiropractic College (CMCC). This educational intervention was presented in Toronto and Calgary in 2005. Testing, survey and demographic data were collected as a routine matter during the workshop for quality improvement purposes. The participants gave oral informed consent to the collection of test, survey and demographic data. They were assured of anonymity and confidentiality. The Research and Ethics Board of the CMCC approved the study protocol.

### Outcome measures

The most widely used and popular model for the evaluation of training and learning is Kirkpatrick's four-level model [29].

The four levels of Kirkpatrick's evaluation model measure:

1. reactions of students – what they thought and felt about the training;

2. learning – the resulting increase in knowledge or capability;

3. performance – extent of behavior and capability improvement and implementation/application; and

4. impact – the effects on the business or environment resulting from the trainee's performance.

We measured learning and participant perception of the quality of the workshop presentation following Kirkpatrick's model for evaluation of educational activities [29]. The primary outcome measures were written tests of EBHC knowledge. We assessed participant self-reported workshop perception. We also collected basic demographic data in writing and asked what peer reviewed journals they regularly read. The phrase "regularly read" was defined by each respondent.

Two measures were developed to test fundamental knowledge of evidence-based methods: a 15-item pretest-posttest measure and a 59-item comprehensive post -intervention measure. The 15-item measure established a baseline for participants' knowledge and aided in motivating participants during the training. The 15-item measure was also nested in the 59-item comprehensive post-intervention measure.

The knowledge measure questions were developed from a pool of 86 items linked to the application of EBHC [30-35]. Considering the practical application of EBHC to chiropractic practitioners, two chiropractic researchers with clinical experience reduced the pool to 59 items for a comprehensive post-intervention test, and 15 of those items were used for the pretest-posttest using a modified Delphi Method. The knowledge measures were tested for readability and comprehensibility in previous workshops. The suggestions of test participants were incorporated to improve the measures' readability and meaningfulness. Content for the knowledge measures paralleled course objectives. The measures employed multiple choice, dichotomized response and free text responses (15-item measure: 14 free text and 1 dichotomized response; 59-item measure: 15 multiple choice, 30 dichotomized responses, 14 free text responses).

For grading purposes, 1 point was allotted per correctly answered question item (partial credit was given for some question items that required multiple responses). Scores were created by converting raw scores to a 0–100 measurement scale by summing the correct items, dividing by the maximum possible total score and multiplying by 100. Participants were given 60 minutes to complete the comprehensive measure; they were given 10 minutes for the 15-item pretest. Participants were not allowed to refer to materials. Exams were self-graded and submitted anonymously. A random sample of tests was examined, and no discrepancies were found between the self-reported scores and instructor scores.

We used four items to measure participants' perception of the presentation (overall evaluation, recommendation of this workshop, knowledge of the presenter and knowledge gained). The response scale for each item was a 5-point scale (e.g., excellent, very good, good, fair, poor). Additionally, we asked participants if the workshop met their learning objectives with a dichotomized scale and allowed for free text comments.

### Educational intervention

We used adult learning theory principles in designing the context of this workshop: the establishment of a need to comprehend, self-direction, task-centered approach, opportunity to receive and offer feedback and attention to the importance of real-life conditions [12,36,37]. We were influenced by continuing education methods that have been shown to have a reasonable chance of influencing behavior in practice: we limited didactic lecture, engaged participants (non-threatening atmosphere), posed provocative questions, acknowledged limitations, applied adult retention schemes for key points and kindled interaction among participants [22]. The instructor, a chiropractic practitioner for 20 years, has been involved in teaching EBHC for almost a decade and is an active researcher and peer reviewer for medical and chiropractic journals.

The aim of the workshop was to develop competency in evidence-based principles and procedures. The overriding goal was for participants to attain the skills to properly integrate scientific evidence by learning the following competencies: (1) articulate clinically important questions; (2) locate and access relevant literature to address the questions; (3) critically appraise the literature for its usefulness and validity; and, (4) utilize the results of assessments to improve patient management [32]. We provided all participants with a 110-page workbook and conducted a 12.5 hour training over a two day period. Group discussion was encouraged, and participants completed worksheets and answered questions within each section to review key concepts. The most critical part of the workshop was interpretation of study results for enhanced patient management.

### Data collection and statistical analysis

We calculated descriptive statistics: percentages, mean, standard deviation and range, as appropriate. For comparative analysis, effect size was estimated [effect size = delta (mean change)/sigma (the standard deviation at baseline]. This study used *Cohen's d *for dependent means effect size method [38]. The following standards were used to interpret effect size data: small size effect (.2); medium size effect (.5); and large size effect (≥ .8) [39]. A positive score indicates knowledge gain, and a negative score indicates knowledge deterioration. Pearson's product moment correlation coefficient was used to test the correlation between knowledge scores and participants' ages and years in practice. All data were entered into a data base and analyzed with Minitab 10.51 Xtra (State College, Pennsylvania). All the data were randomly checked for accuracy.

## Results

### Participants' characteristics

Baseline characteristics of the participants are presented in Table 1. Forty-seven practitioners attended the workshop, and forty provided data for this study (response rate was 85%). The respondents were mostly male (76%) graduates of CMCC (58%) with a mean age of 36 years, approximately 9 years of clinical experience, and most were in solo practice (65%). Eighty-eight percent were enrolled in the Chiropractic Rehabilitation Sciences program at CMCC, and 68% read no peer reviewed journals on a regular basis. In comparison, a random national survey of Canadian chiropractors found that most were male (81%) graduates of CMCC (73%) with a mean age of 40 years and approximately 13 years of clinical experience [40].

**Table 1:** Participant characteristics at baseline (n = 40)

*Characteristic*	
Male, % (n)	76% (31)
Age, mean	36.3 (SD 6.0, min to max 30 – 53)
Years in practice, mean	8.5 (SD 6.5, min to max 2 – 28)
Chiropractic College, % (n)	
CMCC	58% (23)
Palmer	13% (5)
National	10% (4)
Logan	8% (3)
Palmer-West, LACC, New York, Parker	1 participant each
Postgraduate certification, % (n)	
in progress	88% (35)
yes	10% (4)
no	2% (1)
Solo practice, % (n)	65% (26)
Peer Reviewed Journals (read regularly), % (n)*	
none	68% (27)
*JMPT*	15% (6)
*JCCA*	15% (6)
*Spine*	5% (2)
*NEJM*	2% (1)
Cochrane	2% (1)
J Sports Chiro & Rehab	2% (1)

* Participants could select more than one journal.

### Knowledge measures

The mean baseline score for the 15-item pretest was 7.1 (47%) correct responses (min. and max. 2 – 13, SD 2.7). After 11 hours of teaching (over a two-day period), we retested the same participants by nesting the original 15-item test (posttest) into our final exam of 59 questions. The mean score for the posttest was 12.5 (83%) correct responses (min. and max. 8 – 15, SD 1.8). The effect size was 2.0. The mean score for the final exam (59 questions) was 52.1 (88%) correct responses (min. and max. 43 – 59, SD 3.4). Pearson's coefficient produced the following correlations: years in practice correlated very weakly and inversely with pretest score (-.18; P = .37); participant age correlated weakly and inversely with pretest scores (-.34; P = .09). Correlations between years in practice or participant age and post workshop test scores were less than .10 (P > .65).

### Quality of presentation

**Figure 1:**
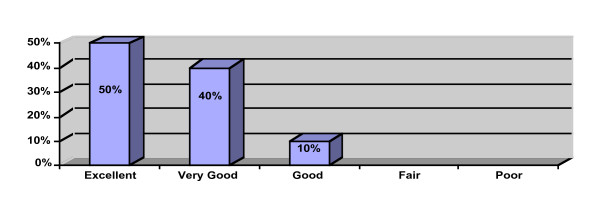
What is your overall evaluation of this workshop (n = 40)?.

**Figure 2:**
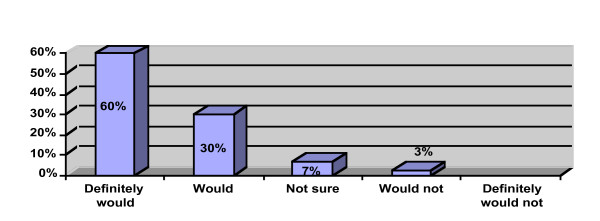
Would you recommend this workshop to a colleague (n = 40)?.

**Figure 3:**
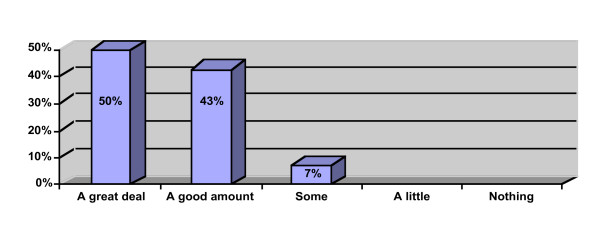
How much knowledge did you gain from this workshop (n = 40)?.

Participant perception data is provided in Figures 1, 2, 3. Ninety percent of the participants rated the workshop overall as "excellent" or "very good" and would "definitely recommend" or "recommend" the workshop to a colleague. All of the participants reported that the workshop met their learning objectives and that the presenter was "very knowledgeable" or "above average" in knowledge. Ninety-three percent reported that they learned "a great deal" or "a good amount" of knowledge. Ten participants provided free text comments that were mostly very positive (Table 2).

**Table 2:** Participant comments (n = 10)

#1. "Excellent! You kept the audience engaged throughout!"
#2. "Fabulous."
#3. "Great presentation. Perhaps add PowerPoint?"
#4. "Made a boring subject interesting."
#5. "Did an excellent job on a very dry topic!"
#6. "Extremely helpful."
#7. "It has changed my ability."
#8. "Engaging. Made a very dry subject enjoyable. Thanks."
#9. "Excellent way to engage adult learners."
#10. "Too much information, very long. Good information. Give information before hand. No reason for people to read any notes out loud."

## Discussion

Evidence-based practitioners are the future leaders of chiropractic, because they possess the ability to apply research and are likely to deliver more effective and less costly interventions. The abilities to assess data critically, evaluate study methods, and evaluate study outcomes are essential cognitive competencies used by successful health care practitioners in caring for their patients in this era of accountability and continual quality improvement. Like most cognitive skills, EBHC principles need to be reinforced through application and repetition [15]. Noted experts have straightforwardly stated that chiropractic practitioners using the EBHC approach benefit from being able to:

*1. Be more effective and efficient*.

*2. Deal with expanded demands for accountability*.

*3. Manage rapidly expanding sources of information*.

*4. Maintain professional competence*.

*5. Provide an increased quality of care *[41].

The results of this study suggest that our educational intervention enhanced practitioners' EBHC knowledge. Workshop participants reported high scores for the quality of the workshop presentation.

The response rate to the surveys and tests was good. Overall, these participants might be considered more interested in research evidence and peer reviewed journals than the average chiropractor. However, more than two-thirds of the participants read no peer reviewed journals on a regular basis. This is not unexpected, because subscription rates to peer reviewed journals are low within the profession.

As expected, participants scored poorly before the workshop intervention and performed exceedingly well in post intervention testing. For the 15-item pre-posttest, the effect size was very large. A mean score of 88% on the 59-item test is considered noteworthy. We compared baseline knowledge scores with participants' ages and found a weak inverse relationship. Also, we compared baseline knowledge scores with participants' years in practice and found a very weak inverse relationship. These findings are not discordant with indecisive findings from a systematic review that reported that medical physicians who have been in practice longer may be at risk for providing lower-quality care and posses less up-to-date knowledge [42]. Posttest scores measuring knowledge gain likewise were not related to participant age or years in practice (i.e., older participants grasped the EBHC concepts just as well as the younger participants).

The quality of the presentation is an important variable in the education process. In this study, participants rated the quality of presentation very high. Free text comments primarily indicated appreciation of the preparation and implementation of the workshop. Because of the anonymity with which data were collected, participants were likely to be honest in their feedback. It is difficult to imagine a poor quality presentation yielding respectable knowledge gains, improving practitioner behavior or improving patient outcomes [2,29]. Thus, participant perception of the presentation's quality is an important element in educational courses.

Our workshop's effectiveness, we believe, derives largely from its fidelity to adult learning theory and implementation of effective health care CE strategies. Educational interventions involving active participant involvement lead to "deeper processing of information" and, consequently, improved recollection of factual information [43]. Interactive educational meetings (participation of healthcare providers in workshops that include discussion and/or practice) are generally effective [2,44]. Passive dissemination of information (e.g., chiefly didactic lecture) is generally ineffective.

### Limitations

The research design used for this study limits inferences that should be drawn from the study's conclusions. This study is a before-after design without controls and is not as strong as a randomized trial. Participants were self-selected, and were possibly more motivated and more likely to benefit from the intervention than the general population of chiropractors.

Our study may also be criticized for reporting on process measures (knowledge gained, quality of presentation), rather than practitioner behavior and clinical outcomes. The long-term goal of continuing education is not merely to impart new knowledge, but rather to change learners' behaviors [45]. However, patient outcomes are difficult to measure and are affected by many other unrelated variables [45]. Measures farther away from the intervention can easily be influenced by un-measurable untoward events compared with the more proximal and easier-to-measure items of competence: knowledge, skills and attitudes. Ideal outcomes would include estimates of a continuum of measures: competence to performance to health care outcomes [2]. Also, our evaluation was short-term, so we cannot assess the durability of the knowledge.

Measurement bias might have occurred, because our test to measure EBHC knowledge had not been previously assessed. However, the impressive improvement between pre- and post-testing scores provides support for the instrument's psychometric qualities. The educational literature recognizes that pre-assessment may lead to a higher post-assessment score due to an item-practice effect [46]. There is a possibility of a test-training effect, because we used an identical posttest; that is, it is possible that participants learned how to take the same test better the second time around, rather than really learning new knowledge. However, participants' scores for the 59-item test were high. It is unlikely that participants inflated their test scores, because they submitted their scores anonymously, and we found no differences between self-reported scoring and instructor scoring.

Future studies should incorporate more rigorous design, including validation of outcome measures, larger sample size, use of a control group, and follow-up. Ideally, upcoming studies should measure the effect of the intervention on patient outcomes, preferably in randomized controlled trials. It is difficult to predict the generalisability of our findings; it is unknown whether other instructors with different content and context would achieve similar improvements [47,48]. Despite these limitations, our evaluation demonstrated a positive impact on the participants' EBHC knowledge and a high perception of the workshop presentation's quality.

## Conclusion

The results of the study imply that the intervention was effective in enhancing EBHC knowledge. The effect size was large. Because there were several potential sources of variation, further study is required. The quality of presentation was graded very highly. Thus, this workshop may provide a model by which EBHC knowledge can be taught to practitioners.

## Competing interests

The author(s) declare that they have no competing interests.

## Authors' contributions

RJF conceived of the study and participated in its design. He also acquired data, analyzed and interpreted data and helped to draft the manuscript. JPG participated in study coordination and interpretation of data and helped to draft the manuscript. ATV participated in study design and was involved in revising the manuscript. All authors read and approved the final manuscript.
